# Metabolomics and Machine Learning Uncover 5′-Methylthioadenosine as a Metabolic Indicator of Mixed-Pattern Drug-Induced Liver Injury

**DOI:** 10.3390/metabo16090614

**Published:** 2026-08-27

**Authors:** Jinghui Zhang, Hongchuan Liu, Qingrong Qiu, Kongcai Zhu, Xian Ding, Rui Zhao, Ting Hu, Zhuoling An

**Affiliations:** 1 Beijing Chao-Yang Hospital, Capital Medical University, Beijing 100020, China; 2 Beijing Youan Hospital, Capital Medical University, Beijing 100069, China

**Keywords:** drug-induced liver injury, metabolomics, 5′-methylthioadenosine, machine learning, biomarker

## Abstract

Background/Objectives: Drug-induced liver injury (DILI) remains a leading contributor to acute liver failure, yet phenotypic classification still relies on the R-value, leaving the mixed-pattern (MD) subtype a heterogeneous category between hepatocellular (HD) and cholestatic (CD) injury. We aimed to identify a metabolic signature that helps resolve this heterogeneity. Methods: Targeted LC-MS/MS metabolomics was performed on 79 patients with DILI (HD = 54, CD = 7, MD = 18) and 221 healthy controls. A 32-metabolite panel was used to train an XGBoost classifier with pre-specified, fold-invariant hyperparameters, evaluated via Leave-One-Out Cross-Validation and interpreted by TreeExplainer-based SHAP attribution. Results: SHAP identified 5′-methylthioadenosine (MTA) as the dominant contributor. A four-parameter logistic fit of its SHAP dependence curve gave an exploratory, model-derived transition point at a relative abundance of 93. Applying this value to the 18 held-out MD patients assigned 16 to a hepatocellular and 2 to a cholestatic trajectory. MTA correlated with total bilirubin (r = 0.53, *p* < 0.001) but not ALT (r = −0.19, *p* = 0.10), dissociating it from hepatocyte leakage. Conclusions: MTA emerges as an exploratory biomarker that uncovers the distinct metabolic trajectories hidden within mixed-pattern DILI. Furthermore, these findings highlight the MTA-methylthioadenosine phosphorylase (MTAP) axis as a potential mechanistic driver of cholestatic injury.

## 1. Introduction

Drug-induced liver injury (DILI) is a leading cause of acute liver failure in the United States and Europe and a frequent reason for post-marketing withdrawal of approved pharmaceuticals [1], and prescription-drug surveillance indicates an incidence high enough to generate frequent unscheduled hepatology consultations and regulatory actions [2]. Culprit agents differ by region: a nationwide analysis of more than 25,000 hospitalised patients in mainland China attributed a substantial share of DILI to traditional Chinese medicines and herbal or dietary supplements [3]. The 79-patient series analysed here was therefore restricted to traditional Chinese medicine (TCM)-associated injury, to control for etiological variance and minimise confounding metabolic noise.

The AASLD [4], ACG [5] and EASL [6] guidelines stratify DILI into hepatocellular (HD; R ≥ 5), cholestatic (CD; R ≤ 2) and mixed-pattern (MD; 2 < R < 5) injury using the R-value, R = (ALT/ULN)/(ALP/ULN) [7]. The R-value relies on two enzymes with disparate temporal kinetics: ALT clears from serum in approximately 47 h, whereas ALP clearance extends across roughly seven days, so the same patient can cross phenotype boundaries depending on the day of sampling—an uncertainty greatest in the mixed-pattern stratum [8]. Meta-analysis has documented prognostic overlap between HD and CD trajectories that the R-cutoff does not resolve [9], and operator variability in RUCAM-based scoring compounds the problem [10]. The consequence is direct: hepatocellular-pattern injury is often managed with glucocorticoids and cholestatic-pattern disease with ursodeoxycholic acid, and the two carry measurably different chronicity risk [11], yet patients with 2 < R < 5 are assigned to either arm without molecular evidence, and the histopathological correlation of the MD label is poor [12,13]. As currently defined, MD therefore behaves as a heterogeneous label that may conflate molecularly distinct entities.

The biomarker landscape remains incomplete. Serum markers such as miR-122, glutamate dehydrogenase and keratin-18 [14,15,16] index the consequences of hepatocyte injury rather than the molecular driver of phenotype and have not redrawn the HD–CD boundary [17]. Targeted serum metabolomics is, by contrast, a sensitive, mechanistically informative layer for disease characterisation [18] and for DILI in particular [19,20,21]. Within current panels, the methionine salvage pathway is an under-explored node of potential cholestatic relevance: MTA is the obligate side-product of polyamine biosynthesis, and its intracellular pool is normally held low by methylthioadenosine phosphorylase (MTAP) [22,23], while the SAM/MTA control switch has been tied to liver function and bile-acid biology [24]. Translating such high-dimensional metabolomic profiles into clinically interpretable decision variables requires robust computational bridging. Tree-based gradient boosting, paired with Shapley-value (SHAP) attribution [25,26], meets the interpretability demands of high-stakes clinical modelling [27] and is increasingly utilised for disease reclassification [28,29].

We hypothesised that a non-linear model applied to a 32-metabolite serum panel would isolate a dominant biochemical signature distinguishing pure hepatocellular from pure cholestatic DILI that persists across the heterogeneity of the mixed-pattern label. After selecting a discriminant feature set, we trained an XGBoost classifier exclusively on the unambiguous phenotypes. TreeExplainer-based SHAP attribution was utilised to identify the dominant feature and derive an analytical, single-molecule transition point. The trained classifier was subsequently applied—without retraining—to 18 clinically labelled MD patients as an independent test set to determine their molecular trajectories. Finally, we synthesised this cohort-level evidence into a hypothesis-generating framework centred on the MTA–MTAP axis, proposing MTA as a candidate molecular indicator that complements the empirical R-value, pending prospective external validation.

## 2. Materials and Methods

### 2.1. Study Design, Cohort and Ethics

This observational study included a clinical cohort of 79 patients with DILI alongside 221 healthy controls. To strictly control for sample variance, enrolment was exclusively restricted to cases attributable to TCM. For brevity, this TCM-restricted cohort is referred to simply as DILI throughout the remainder of this text. Patient enrolment was conducted at two clinical centres: cases at Beijing Youan Hospital were identified between April 2015 and April 2019 under an institutional waiver of informed consent for the use of retrospective clinical records. The reference cohort of 221 healthy volunteers was recruited from the physical examination centre of Beijing Chao-Yang Hospital between April 2018 and April 2019.

To ensure unambiguous phenotypic stratification, DILI cohort inclusion was strictly limited to patients with complete initial biochemical profiles permitting clinical R-value calculation. Consequently, the 79 enrolled DILI patients were classified into hepatocellular (HD, *n* = 54), cholestatic (CD, *n* = 7), and mixed-pattern (MD, *n* = 18) subtypes. Routine diagnostic workup included rigorous imaging-based exclusion of extrahepatic biliary obstruction (via abdominal ultrasound, supplemented by computed tomography or magnetic resonance cholangiopancreatography where clinically indicated), in adherence to AASLD and EASL diagnostic recommendations; patients with evidence of mechanical obstruction were excluded. The study protocol was approved by the Ethics Committees of Beijing Youan Hospital (LL-2019-155-K) and Beijing Chao-Yang Hospital (2017-Research-150).

### 2.2. Inclusion and Exclusion Criteria

Adult patients (≥18 years) with a documented history of ingestion of TCM and onset of digestive symptoms (anorexia, fatigue, upper-abdominal discomfort, nausea or vomiting) within one to four weeks of exposure were considered eligible. Liver-function abnormalities had to be present (ALT, AST, ALP or TBIL above the sex-specific upper limits of normal) and the diagnosis had to meet the criteria for DILI under the AASLD [4] and ACG [5] frameworks. To ensure the etiological purity of the cohort, rigorous exclusion criteria were implemented. Patients were excluded if they presented with competing causes of liver injury, including viral hepatitis (HBV, HCV, or other infectious hepatitis), autoimmune hepatitis, alcoholic or non-alcoholic fatty liver disease, hereditary metabolic liver diseases (such as haemochromatosis, Wilson disease, or α1-antitrypsin deficiency), and cirrhosis. Additional exclusion criteria comprised concomitant diabetes mellitus, gout, phenylketonuria, current or previous malignancies, pregnancy or breastfeeding, a history of organ transplantation, undocumented culprit-drug records, or severe concurrent systemic diseases. Phenotypic classification was determined using the R-value, calculated as R = (ALT/ULN)/(ALP/ULN), with sex-specific upper limits of normal applied to both enzymes. In accordance with the standardised DILI phenotype framework [7] and the Chinese guideline for the diagnosis and treatment of DILI [30], patients were stratified into hepatocellular (HD, R ≥ 5), cholestatic (CD, R ≤ 2), and mixed-pattern (MD, 2 < R < 5) subtypes.

### 2.3. Serum Sampling and Targeted LC-MS/MS Metabolomics

Targeted quantitative metabolomics analysis was performed following the previously reported method [31]. Serum aliquots were acquired during routine diagnostic phlebotomy. Samples were allowed to coagulate at 4 °C, followed by centrifugation (3000 rpm, 10 min, 4 °C), and the resulting supernatants were cryopreserved at −80 °C prior to extraction. Metabolites were extracted via methanol-induced protein precipitation, and the metabolic profiles were acquired using a targeted liquid chromatography-tandem mass spectrometry (LC-MS/MS) platform operating in dual-polarity mode. The key analytical parameters are summarised here for accessibility: the panel comprised 143 metabolites acquired in a single scheduled multiple-reaction-monitoring injection in both polarities on an AB Sciex API 5500 triple-quadrupole instrument (AB Sciex, Framingham, MA, USA); pooled-serum quality-control (QC) samples were injected at a frequency of one QC per 20 study samples; and the inter-day relative standard deviation of every reported metabolite in the pooled QCs was below 20%, consistent with community recommendations for quality control in mass-spectrometry-based clinical metabolomics [32]. The comprehensive methodological details, including reagent specifications, chromatographic gradients, mass spectrometry parameters, and quality control procedures, are fully documented in Supplementary Methods S1.

### 2.4. Differential Metabolite Selection

To comprehensively capture both global hepatotoxicity markers and sub-phenotype-specific metabolic signatures, differential feature selection was performed under a union strategy. The healthy control cohort (n = 221) was contrasted against the overall DILI cohort (n = 79) and against each DILI sub-phenotype (HD, CD and MD) individually. A metabolite was retained if it satisfied the composite criteria in any of these pairwise comparisons: variable importance in projection (VIP) > 1.0 in the respective OPLS-DA model, a two-sided Mann–Whitney U test *p* < 0.05, and a fold change above 1.2 or below 0.8. 32 metabolites passed at least one of these comparisons and were carried forward as the input feature matrix for all downstream OPLS-DA, machine-learning, SHAP and pathway analyses. All retained metabolites robustly satisfied the univariate significance threshold (*p* < 0.05) in their respective inclusion contrasts; their exact VIP scores, log_2_ fold changes, and the specific comparisons driving their selection are detailed in Supplementary Table S1.

### 2.5. OPLS-DA and KEGG Pathway Enrichment

OPLS-DA models were fitted on the 32 retained metabolites in two configurations [33,34]: healthy versus DILI, and healthy versus the three R-defined DILI sub-phenotypes (HD, CD, MD). VIP scores and log_2_ fold changes were rendered as horizontal bar charts in which bar length encodes VIP and a continuous colour gradient encodes log_2_ fold change. KEGG-style pathway enrichment was performed for the same 32 metabolites in MetaboAnalyst (version 6.0) [35]. The 32 differential metabolites were submitted as common compound names and mapped to the platform’s internal identifiers. Enrichment ratios and *p* values were derived via over-representation analysis against the KEGG-derived Homo sapiens metabolite set library, which served as the default background reference metabolome in MetaboAnalyst (version 6.0). The 25 top-ranked sets were rendered as a bubble plot in which dot size encodes enrichment ratio and dot colour encodes the enrichment *p* value.

### 2.6. XGBoost Classifier with Within-Fold SMOTE Under Leave-One-Out Cross-Validation

An eXtreme Gradient Boosting (XGBoost) classifier [36] was trained on the 61 patients with clear R-value phenotypes (HD = 54, CD = 7) using the 32-metabolite feature matrix. To address the inherent class imbalance caused by the epidemiological scarcity of CD, Leave-One-Out Cross-Validation (LOOCV) was implemented. Synthetic Minority Over-sampling Technique (SMOTE) [37,38] was applied exclusively within each training partition before model fitting, ensuring that the held-out validation sample was never seen during any stage of training, hyperparameter selection, or synthetic data generation. Standardised feature scaling (z-score) was performed within each LOOCV training fold. Hyperparameters were pre-specified before cross-validation (shallow trees, max_depth = 3; learning rate = 0.05; 50 estimators; colsample_bytree = 0.80) and held fixed across all 61 folds. No nested or fold-specific tuning was performed (Supplementary Methods S3). This design prevents optimistically biased performance estimates in small cohorts where variance cannot be reliably estimated [39]. Consequently, no information from the held-out samples leaked into feature scaling, synthetic-sample generation, or model configuration. Within each training partition, SMOTE (imbalanced-learn, k_neighbors = 2) generated minority-class samples exclusively via linear interpolation between an observed cholestatic sample and its nearest neighbours in the standardised 32-dimensional feature space. No stochastic noise was added, and no samples were duplicated. Therefore, every synthetic point lies inside the convex hull of the observed minority data, strictly bound within empirical metabolite ranges. The parameter k_neighbors was reduced from 5 to 2 because each training partition contained only six cholestatic cases, preventing interpolation across unsupported feature space. While this densifies the minority class, it cannot diversify it [40]. Model development followed TRIPOD+AI recommendations [41].

### 2.7. SHAP-Based Interpretability and Four-Parameter Logistic Threshold

To decode the decision boundary of the trained XGBoost classifier, SHapley Additive exPlanations (SHAP) attributions were computed using TreeExplainer, which provides exact, locally accurate and consistent additive feature attributions for tree ensembles [26]. To preserve clinical fidelity, SHAP values were projected exclusively onto the real definitive cohort (n = 61); samples generated by SMOTE were not used for attribution. Global feature importance was summarised as the mean absolute SHAP value per metabolite, and individual contributions were inspected by SHAP dependence plots. For MTA, the non-linear dependence between relative abundance x and SHAP value y was modelled by a four-parameter logistic (4PL) regression. Parameters were estimated by non-linear least squares optimisation with the Levenberg–Marquardt algorithm. The 95% confidence band of the fit was generated analytically by Monte Carlo sampling (*n* = 1000) from a multivariate normal distribution defined by the optimised parameter vector and its covariance matrix, propagating each draw through the 4PL function and summarising percentile envelopes on a dense abundance grid. The reference cutoff of MTA was defined as the relative abundance at which the fitted curve crossed SHAP = 0 and was obtained analytically by inversion of the 4PL equation. Full derivations, convergence diagnostics and Monte Carlo procedures are in Supplementary Methods S2.

### 2.8. Metabolic Trajectory Assignment of MD Patients

The trained XGBoost ensemble was applied to the 18 MD patients to compute the predicted probability of the cholestatic phenotype, *P*(CD), for each individual. Given that the training prior was balanced via SMOTE, a pre-specified decision threshold of *P*(CD) > 0.50 assigned a patient to the CD metabolic trajectory, while *P*(CD) ≤ 0.50 assigned the patient to the molecular HD trajectory. To explain individual assignments at the single-patient level, TreeExplainer-based SHAP waterfall plots [26] were produced for each MD patient, and the top ten metabolite contributions were displayed individually, with the remaining 22 features aggregated to preserve the additive closure of the Shapley decomposition.

### 2.9. Statistical Analysis

Orthogonal partial least squares discriminant analysis (OPLS-DA) and the calculation of variable importance in projection (VIP) scores were executed using SIMCA (Umeå, Sweden). Statistical computations, robust machine-learning workflows (including XGBoost modelling and SHAP attribution), 4PL non-linear curve fitting with Monte Carlo confidence-band simulations, and data visualisations were performed in Python (version 3.12.13). The core computational dependencies included scikit-learn (v1.6.1), xgboost (v3.2.0), shap (v0.51.0), scipy (v1.16.3), numpy (v2.0.2), pandas (v2.2.2), matplotlib (v3.10.0), and seaborn (v0.13.2).

## 3. Results

### 3.1. Global Metabolic Remodelling and Pathway Architecture of DILI

**Figure 1:**
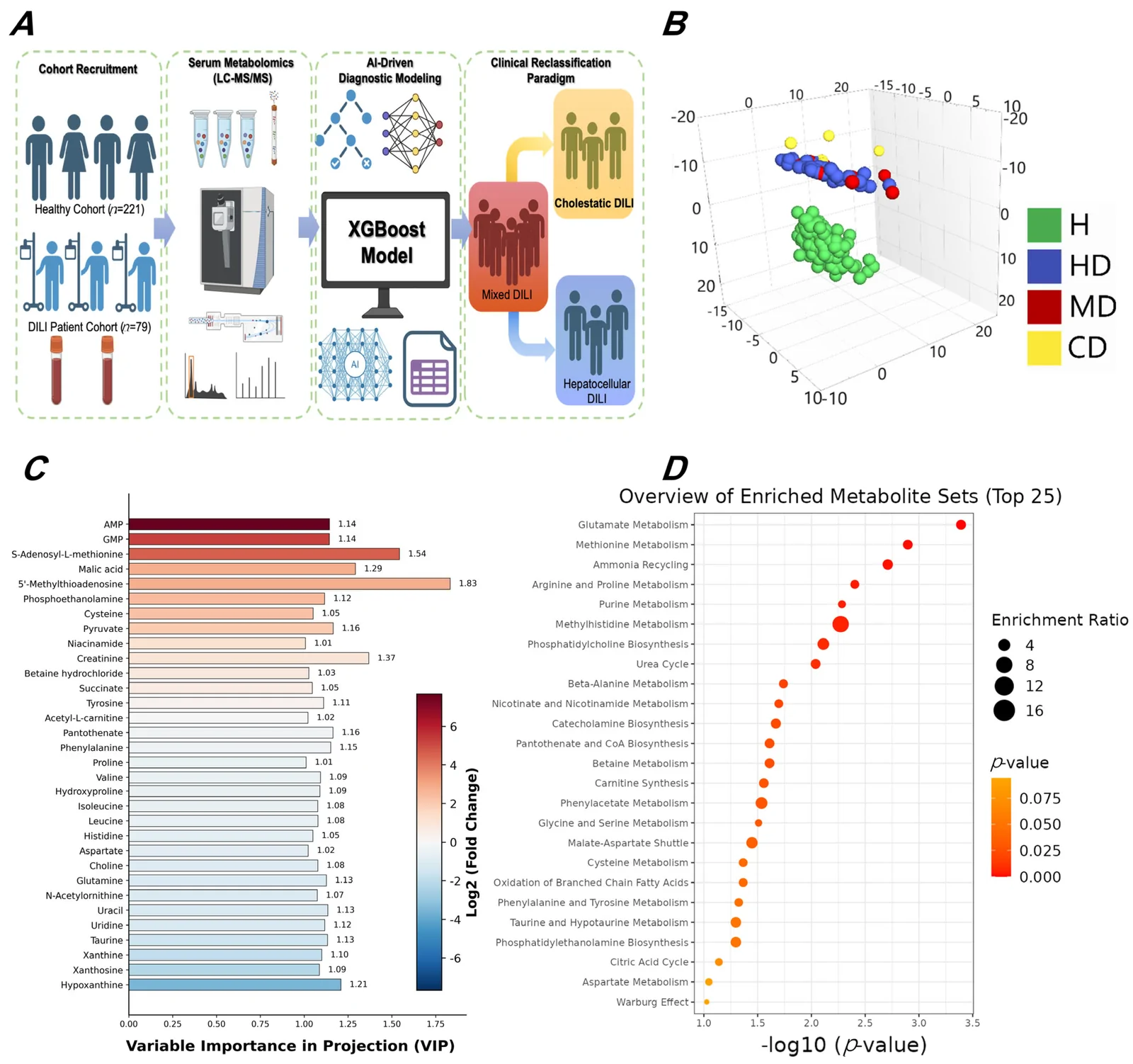
Study design and global metabolic landscape of drug−induced liver injury. (**A**) Analytical workflow. (**B**) Three−dimensional OPLS−DA score plot of healthy controls (H, *n* = 221) and DILI sub−phenotypes (HD, *n* = 54; MD, *n* = 18; CD, *n* = 7). (**C**) VIP−ranked horizontal−bar plot of 32 differential metabolites. Bar length encodes VIP score; colour indicates log_2_ fold change (red: upregulated; blue: downregulated). (**D**) Bubble plot of the top 25 enriched metabolite sets. Dot size reflects the enrichment ratio; colour encodes the enrichment *p* value.

The analytic cohort comprised 79 patients with DILI (HD = 54, CD = 7, MD = 18) and 221 healthy controls. R-value-based classification followed the CIOMS/RUCAM convention [7,42]. Targeted serum metabolomics quantifying 143 metabolites by scheduled MRM LC-MS/MS was performed on every participant, and the analytical workflow is summarised in Figure 1A. Baseline demographic and biochemical features are reported in Table 1. Patients were older than healthy controls (HD 48.0 ± 13.7, CD 56.0 ± 17.9, MD 51.3 ± 13.6 versus 32.3 ± 10.3 years) and predominantly female once injury was established (35.2%, 28.6% and 16.7% male across HD, CD and MD versus 84.6% among controls). ALT, AST, ALP, TBIL and the R-value differed significantly across the four groups, confirming biochemical separation of the three R-defined sub-phenotypes (Table 1).

Differential metabolite selection was performed under the union strategy described in Section 2.4. Of the 143 quantified analytes, 32 met the composite criteria (VIP > 1.0, *p* < 0.05 and fold change > 1.2 or <0.8) in at least one of the contrasts between healthy controls and overall DILI, HD, CD or MD. The full panel and the comparison driving each entry are reported in Supplementary Table S1. OPLS-DA built on this 32-feature matrix (Figure 1B) separated DILI from healthy controls along the first predictive component, but the three DILI sub-phenotypes intermixed within the disease region; linear decomposition was therefore insufficient to resolve HD, CD and MD identity on the global panel.

Ranking the 32 retained metabolites by VIP score and colouring each bar by log_2_ fold change showed a bidirectional metabolic shift between DILI and controls (Figure 1C). A defined subset of features was upregulated relative to healthy levels, consistent with a systemic metabolic stress response after liver injury, while a smaller pool of basal nutrients was reduced. The features extending furthest along the VIP axis correspond to the most heavily weighted metabolic disturbances in the disease state and define the candidate pool subsequently examined under supervised analysis.

KEGG pathway enrichment of the same 32 metabolites assigned the strongest enrichment signal to glutamate metabolism, followed by methionine recycling, methionine metabolism and purine metabolism, with secondary enrichment in polyamine biosynthesis, the urea cycle and arginine and proline metabolism (Figure 1D). The high ranking of glutamate metabolism is biologically expected and reflects the non-specific amino-acid disturbance that accompanies large-scale hepatocyte injury, including the release of transaminases (ALT, AST) and the systemic readjustment of amino-acid pools described in earlier DILI metabolomics work [19]. This component of the enrichment signal therefore reflects a generic necroinflammatory background rather than a sub-phenotype-specific axis. By contrast, the second-ranked enrichment, methionine metabolism, sits at the intersection of methionine recycling and purine metabolism through MTA, the obligate side-product of polyamine biosynthesis and the substrate of the methionine salvage pathway [22]. The convergence of three enriched pathways on this single metabolite identified MTA as a candidate worth examining further, although the question of whether MTA actually carries sub-phenotype-specific information could not be settled from the enrichment map alone and required the supervised analysis developed below.

Taken together, the global view yielded two observations that shaped the remainder of the analysis. The 32-metabolite signature separated DILI from healthy controls (Figure 1B), supporting metabolomics as a sensitive readout of DILI [8,19,43]; the same linear projection, however, did not draw an interpretable boundary between HD, CD and MD, indicating that the latent biology distinguishing the sub-phenotypes was not captured by linear discrimination on the global panel. The analysis therefore proceeded to a non-linear framework targeting the two unambiguous phenotypes before returning to the indeterminate MD group.

### 3.2. Non-Linear Classification on the Unambiguous Phenotypes and a SHAP-Derived 4PL Threshold for MTA

**Figure 2:**
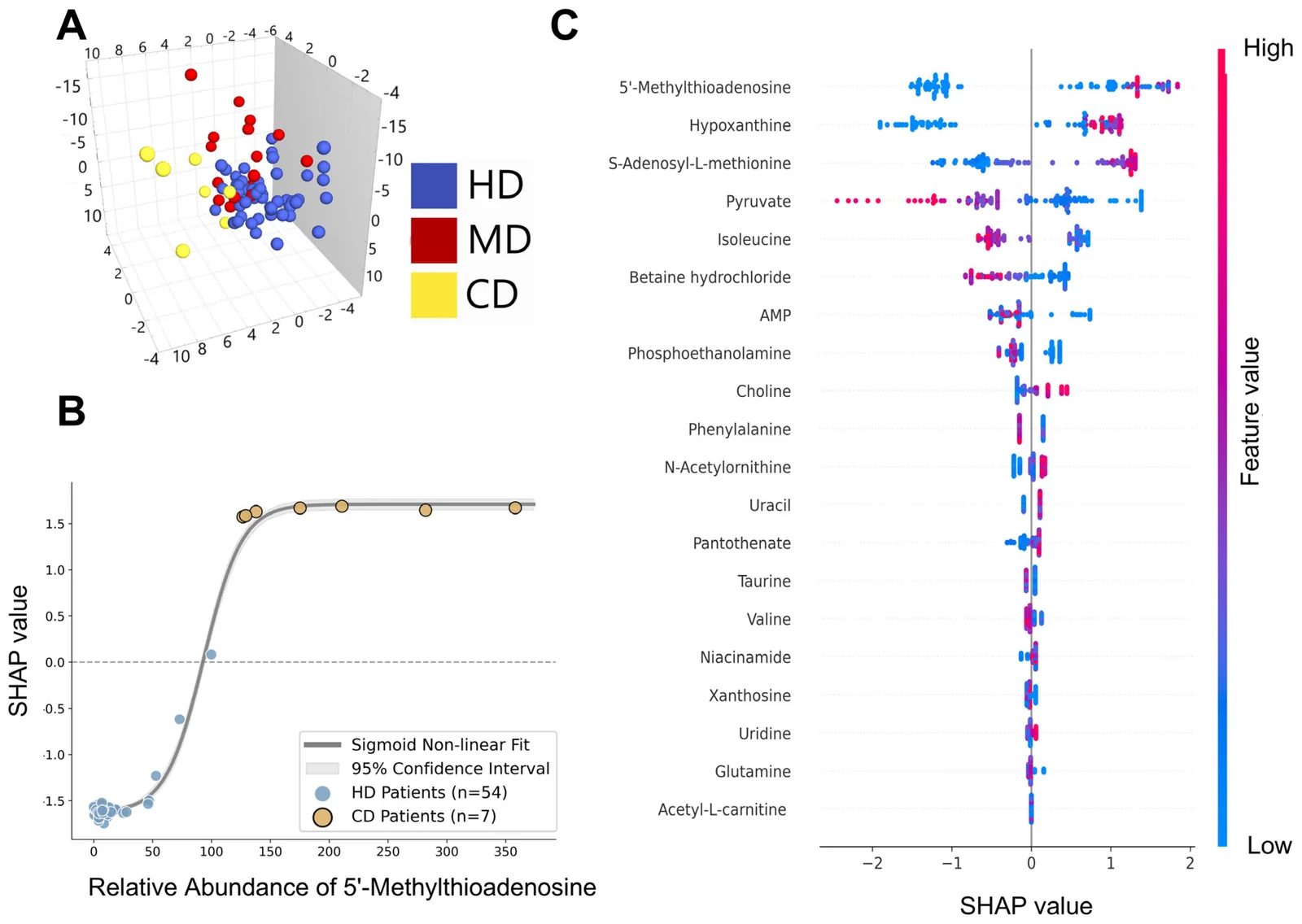
OPLS–DA projection and SHAP–interpreted XGBoost classification of DILI sub–phenotypes. (**A**) Three–dimensional OPLS–DA score plot of the three DILI sub–phenotypes (HD, *n* = 54; MD, *n* = 18; CD, *n* = 7; total *n* = 79). (**B**) Global SHAP summary plot of the top 20 metabolites ranked by mean absolute SHAP value. Each point represents an individual patient. Horizontal position encodes the SHAP value (impact on model output); colour indicates relative metabolite abundance (red, high; blue, low). (**C**) SHAP dependence surface of MTA in the unambiguous DILI cohorts.

When OPLS-DA was restricted to the 79 DILI patients and the 32 differential metabolites, HD, CD and MD point clouds overlapped extensively in score space (Figure 2A). HD and CD share a substantial pool of non-specific necroinflammatory features, whose high variance dominates the linear projection and obscures any sub-phenotype-specific signal. Linear dimensionality reduction was therefore insufficient to separate the two unambiguous phenotypes, and a non-linear classifier capable of decoupling latent sub-phenotype signatures from this shared background was applied next.

Evaluated via a rigorous LOOCV framework to prevent data leakage, the XGBoost classifier achieved a highly robust out-of-sample AUC of 0.950, accurately identifying 54 out of 54 hepatocellular cases and 6 out of 7 cholestatic cases. Given the intrinsically divergent biochemical nature of the pure hepatocellular and pure cholestatic phenotypes, this high discriminatory performance is biologically expected. Rather than serving as an endpoint, this robust classification boundary provided the computational foundation for extracting phenotype-specific mechanistic drivers via SHAP analysis.

To examine which metabolites carried the largest contribution to the trained classifier and to make the model auditable, SHAP attributions were computed by TreeExplainer for every patient [26]. Aggregating absolute SHdAP magnitudes across patients yielded the global feature ranking shown in Figure 2B. The classifier did not distribute weight evenly across the inputs but ranked MTA first by a clear margin over the next-ranked features. MTA therefore emerged as a dominant contributor to the CD prediction.

Plotting per-patient SHAP values against the relative abundance of MTA on the *n* = 61 cohort revealed a sigmoidal rather than linear relationship (Figure 2C). At lower relative abundances, the SHAP values for MTA were consistently negative and clustered the HD patients below the decision baseline (SHAP = 0). Above a defined concentration range, the SHAP values rose steeply and then saturated at a positive plateau for the CD patients. To formalise this transition, a four-parameter logistic function was fitted to the dependence data by Levenberg–Marquardt optimisation, and a 95% confidence band was constructed by Monte Carlo sampling (*n* = 1000) from the optimised parameter covariance matrix. Solving the fitted curve analytically for SHAP = 0 placed the transition at a relative abundance of approximately 93. Below this value, MTA contributed negatively to the CD prediction, and above it the contribution rose onto a positive plateau. The Monte Carlo confidence band around 93 defines a transition region in which the model’s MTA-driven contribution changes sign and shows that MTA enters the CD prediction in a threshold-like rather than a graded fashion. The numerical value of 93 is strictly a model-derived transition point obtained from a single, small cohort using a specific analytical workflow; it is explicitly not a clinical decision limit. Expressed in relative-abundance units specific to the internal standards and normalisation of our LC-MS/MS assay, this value is not directly transferable to other laboratories or platforms. Establishing its reproducibility across different analytical workflows, its stability under independent resampling, and its behaviour in populations with diverse demographics and DILI aetiologies requires prospective external validation. With the model-derived transition region defined, the next analysis tested whether the MTA signal was independent of generic hepatocellular leakage.

### 3.3. Dissociation of MTA from Hepatocellular Necrosis and Coupling to Cholestatic Injury

**Figure 3:**
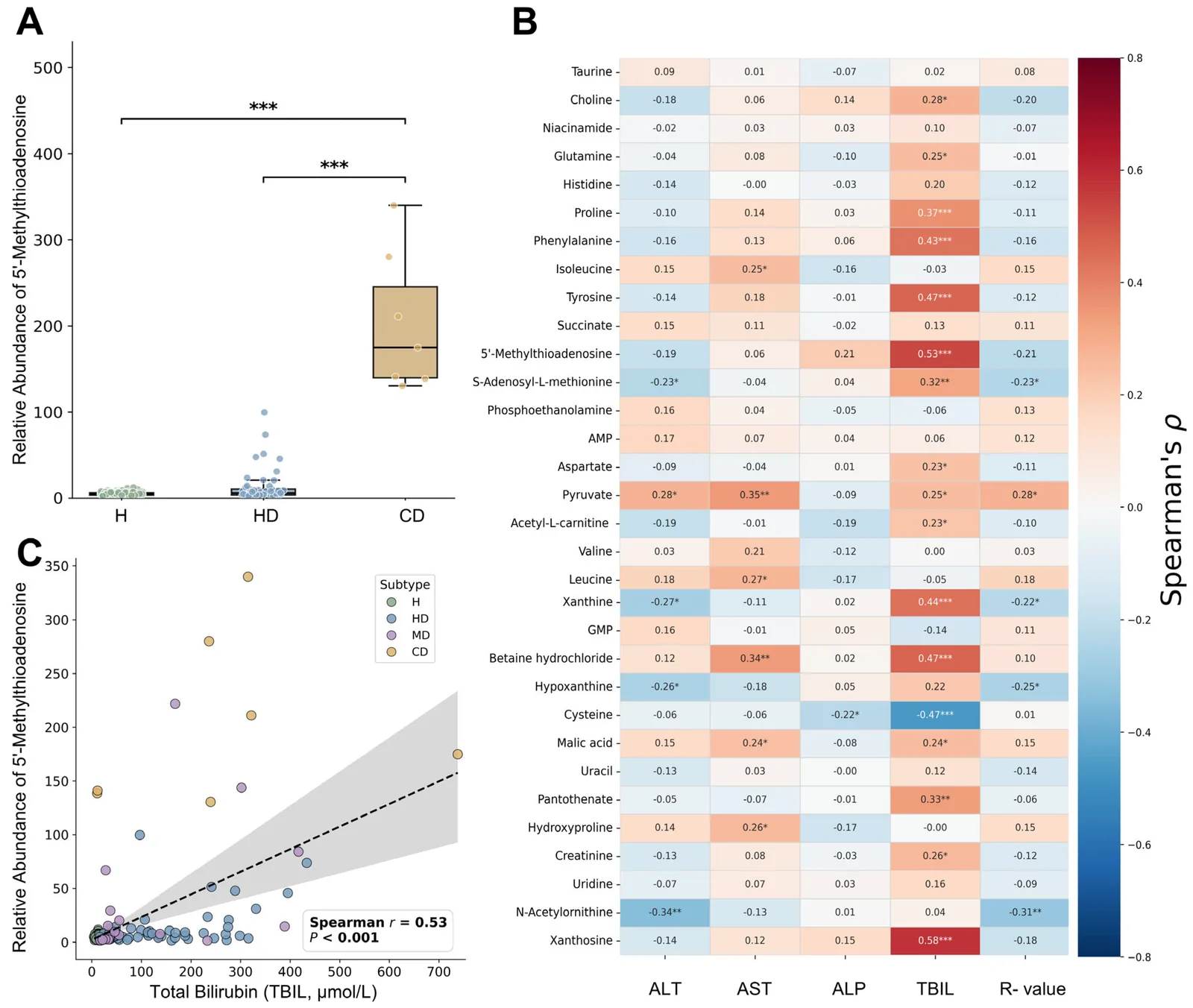
Dissociation of MTA from hepatocellular necrosis and coupling to cholestatic injury. (**A**) Relative MTA abundance across healthy controls (H, *n* = 221), hepatocellular (HD, *n* = 54), and cholestatic (CD, *n* = 7) phenotypes. In the box plot, the central horizontal line represents the median, the box spans the interquartile range. Dots represent individual sample values. *** *p* < 0.001 compared with the CD group. (**B**) Spearman correlation heatmap linking the 32 differential metabolites (rows) to five clinical indices (columns). Cell values denote Spearman’s rank correlation coefficient (*r*), with colour encoding magnitude and direction (blue to red: −0.6 to +0.6). Statistical significance for individual correlation coefficients is annotated as * *p* < 0.05, ** *p* < 0.01, and *** *p* < 0.001. (**C**) Scatter plot correlating relative MTA abundance with serum total bilirubin across all subjects (H: grey; HD: blue; MD: purple; CD: yellow). The dashed black line represents the linear regression fit (r = 0.53, *p* < 0.001), flanked by the 95% confidence interval (shaded band).

Three complementary analyses tested whether MTA tracks generic hepatocellular leakage or a cholestasis-specific excretory failure. Relative MTA abundance was first compared across three clinically defined groups: healthy controls (n = 221), HD (n = 54) and CD (n = 7) (Figure 3A). Relative MTA abundance did not differ significantly between the healthy control and HD groups, notwithstanding that the median serum ALT and AST levels in the HD group were approximately 50- and 30-fold higher than those of healthy controls, respectively (Table 1). Both the HD versus CD and the healthy versus CD comparisons reached *p* < 0.001, with CD patients showing higher MTA values relative to either reference group. The lack of difference between the healthy and HD groups demonstrates that overtly compromised hepatocyte membrane integrity does not increase circulating MTA. Consequently, the MTA elevation observed in CD cannot be attributed to generic hepatocellular leakage.

Spearman correlation between each of the 32 metabolites and five canonical liver-injury indices (ALT, AST, ALP, TBIL and R-value) across all DILI patients reinforced this dissociation (Figure 3B). MTA was positively correlated with total bilirubin (r = 0.53, *p* < 0.001), the established serum marker of biliary excretory dysfunction, but not with the necrosis markers ALT (r = −0.19, *p* = 0.10) or AST (r = 0.06, *p* = 0.61). Xanthosine, a purine metabolite functionally adjacent to MTA, showed a similar coupling with TBIL (r = 0.58, *p* < 0.001), consistent with coordinated nucleotide-pool remodelling during cholestasis. As an internal positive control, pyruvate correlated with ALT (r = 0.28, *p* = 0.01), confirming that the correlation framework correctly resolves a necrosis-coupled signal in the same dataset in which MTA does not track ALT. The full Spearman r and *p*-value matrices for all 32 metabolites are in Supplementary Tables S3 and S4.

At single-patient resolution, relative MTA abundance was plotted against serum TBIL across the entire cohort, colour-coded by subtype, with a linear regression line and 95% confidence band (Figure 3C). The fit confirmed the heatmap result (Spearman r = 0.53, *p* < 0.001), but the scatter geometry was not a uniform diagonal. In the lower-right region of the plot, several severe HD patients with TBIL above 300 μmol/L nevertheless retained basal-range MTA, indicating that hyperbilirubinaemia driven by hepatocellular necrosis is not sufficient to raise MTA in the absence of cholestatic-pattern pathology. Two CD patients in the same scatter, with TBIL within the healthy reference range, nevertheless showed relative MTA values above the 93 transition point; this is consistent with the threshold-like behaviour of MTA in this cohort but is observed in only two patients and is noted as exploratory rather than as evidence for an early-sentinel role. Collectively, these data firmly uncouple MTA from generic hepatocellular necrosis and establish it as a specific correlate of cholestatic injury. Within this framework, the observation that most MD points sat on the HD trajectory at low MTA, while a minority projected upward onto the CD plateau, directly motivated the formal reclassification analysis below.

### 3.4. Metabolic Trajectory Assignment of Clinically Mixed-Pattern DILI in an Independent Test Cohort

The XGBoost classifier described above was trained, cross-validated and SHAP-fitted exclusively on the 61 patients with unambiguous R-value phenotypes (HD = 54, CD = 7). The 18 MD patients with 2 < R < 5 were withheld from every step of model fitting, hyperparameter selection, SMOTE over-sampling and SHAP attribution, and were applied to the trained model only once as a held-out independent test cohort. For each MD patient, the classifier returned the predicted probability *P*(CD), evaluated against the pre-specified decision threshold of 0.50.

**Figure 4:**
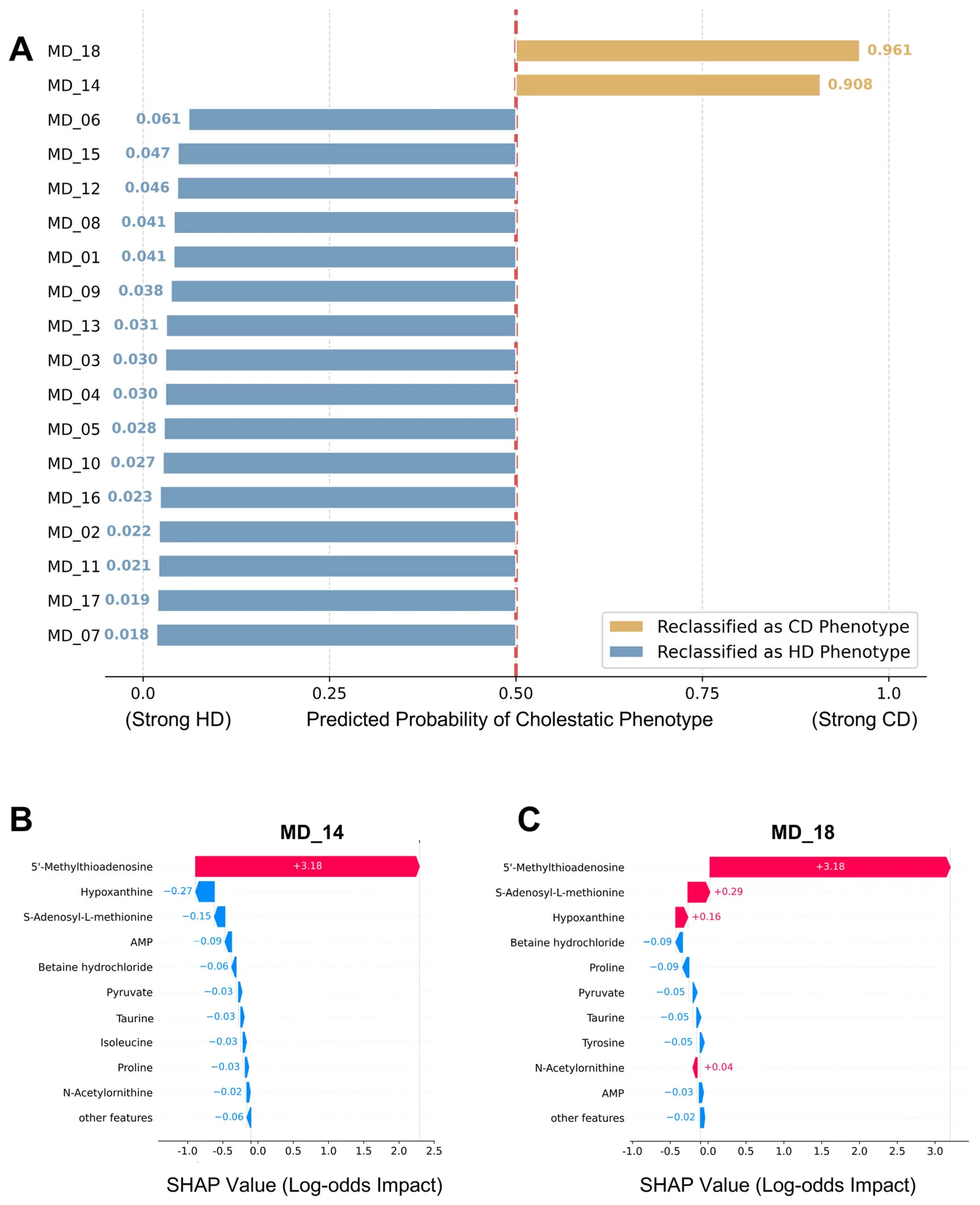
Metabolic trajectory assignment of mixed–pattern DILI. (**A**) Waterfall plot of XGBoost–derived predicted probabilities of the cholestatic phenotype for the 18 MD patients. The cohort is ranked in descending order of predicted *P*(CD); the vertical red dashed line at *p* = 0.50 marks the decision boundary. (**B**) Individualised SHAP waterfall plot for patient MD_14. The horizontal axis represents SHAP values. Red vectors drive the prediction toward CD, while blue vectors oppose it. (**C**) Individualised SHAP waterfall plot for patient MD_18.

Ranking the 18 MD patients in descending order of predicted *P*(CD) produced the waterfall in Figure 4A. Rather than clustering around the 0.50 boundary that the descriptive label of mixed-pattern might suggest, the 18 patients separated into two groups. Two patients showed predicted probabilities well above the threshold, MD_18 with *P*(CD) = 0.961 and MD_14 with *P*(CD) = 0.908, and the remaining 16 patients showed probabilities well below the threshold (*P*(CD) range: 0.018–0.061). The dichotomy reflects both the underlying metabolic signal and a structural property of the model in this cohort. Sixteen of 18 MD patients (88.89%) were placed on the HD-pattern trajectory and 2 of 18 (11.11%) on the CD-pattern trajectory. Within the resolution of the 32-metabolite panel, the R-value MD label was distributed across two distinct metabolic signatures rather than a continuum. Per-patient predicted probabilities are reported in Supplementary Table S5.

To examine the diagnostic call at single-patient resolution, TreeExplainer-based SHAP waterfalls were generated for the two MD patients assigned to the CD metabolic trajectory (Figure 4B, MD_14; Figure 4C, MD_18). The top ten metabolite contributions were displayed individually, and the remaining features were aggregated to preserve the additive closure of the Shapley decomposition. In MD_14, the predicted log-odds were driven by a positive SHAP contribution from MTA of +3.18, with the next-ranked contributions being hypoxanthine (−0.27), S-adenosyl-L-methionine (−0.15), AMP (−0.09) and betaine hydrochloride (−0.06); the antagonistic terms were more than an order of magnitude smaller than the MTA contribution. The same pattern, with greater consistency of sign, was observed in MD_18, where MTA again contributed +3.18 log-odds, reinforced by S-adenosyl-L-methionine (+0.29), hypoxanthine (+0.16) and N-acetylornithine (+0.04), with smaller antagonistic contributions from betaine hydrochloride (−0.09), proline (−0.09), pyruvate (−0.05), taurine (−0.05) and tyrosine (−0.05). In both patients, the residual metabolites aggregated to a near-zero net effect (−0.06 in MD_14 and −0.02 in MD_18). The relative MTA abundance in both patients exceeded the 4PL-derived transition point of approximately 93 (MD_14, 143.74; MD_18, 221.81). Consequently, both values mapped to the same saturated CD plateau of the SHAP dependence curve, yielding the identical maximal log-odds contribution (+3.18) characteristic of rightward terminal leaf nodes in tree ensembles. Although the R-value placed both patients in the same indeterminate zone alongside the other 16 MD cases, the local SHAP decomposition described a CD-pattern metabolic signature dominated by MTA, with all other features contributing minor and largely cancelling perturbations.

### 3.5. A Metabolomics-Derived Hypothesis: Putative MTA–MTAP Axis as the Basis of Cholestatic MTA Accumulation

**Figure 5:**
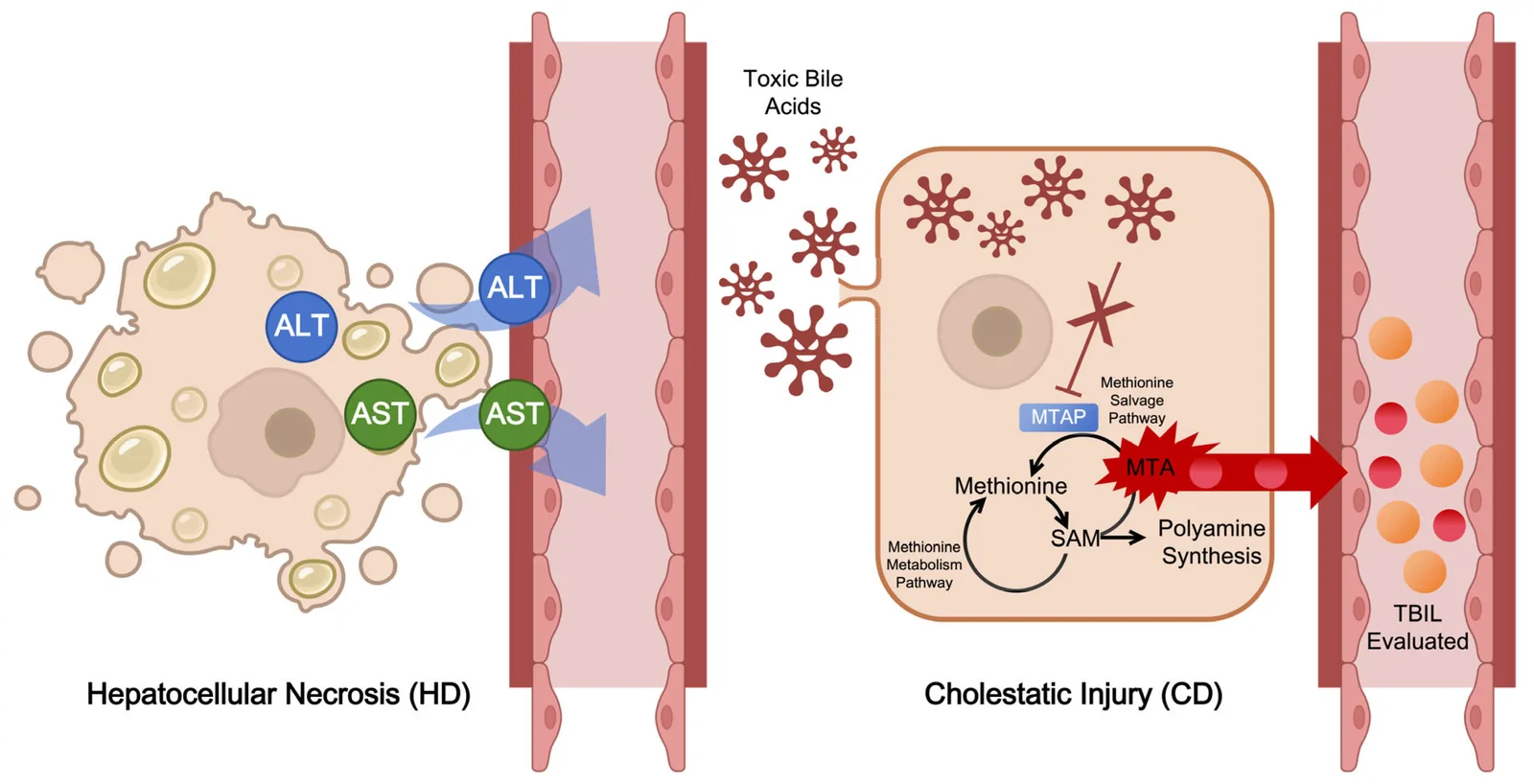
Hypothesis-generating MTA–MTAP framework suggested by the present metabolomic profile.

Synthesising the computational, biochemical and clinical–correlational findings above, we outline a hypothesis-generating mechanistic framework, derived from the present metabolomic profile and put forward for future enzymatic and in vivo testing, that could account for the divergent metabolic fates of MTA between the two distinct DILI phenotypes (Figure 5).

In the HD scenario (Figure 5, left panel), the dominant event is non-specific hepatocyte membrane disintegration. Cytosolic enzymes such as ALT and AST passively spill into the sinusoid, but MTA does not accumulate, consistent with the lack of difference between healthy and HD in Figure 3A and the absence of an MTA–ALT correlation in Figure 3B (r = −0.19, *p* = 0.10). In the CD scenario (Figure 5, right panel), viable but perturbed hepatocytes accumulate toxic hydrophobic bile acids intracellularly driven by an imbalance between continued hepatic uptake and impaired canalicular excretion [44,45,46]. We hypothesise that this bile-acid load impairs or downregulates MTAP within the methionine salvage pathway. Under physiological conditions, MTAP catalyses the phosphorolysis of MTA, the obligate side-product of polyamine biosynthesis, to 5-methylthioribose-1-phosphate, holding the cellular MTA pool at a low steady state [22,23]. Reduced flux through this node would create a metabolic bottleneck that allows MTA to accumulate and spill into the systemic circulation alongside elevated bilirubin, in line with the systemic MTA elevation described in MTAP-deficient cancer biology [47].

Within this framework, we further hypothesise that the model-derived transition point of 93 marks the abundance at which residual MTAP recycling capacity is exceeded. Once this recycling capacity is overwhelmed, the salvage pathway can no longer clear excess MTA, causing circulating MTA to accumulate and eventually plateau. MTAP protein expression, MTAP enzymatic activity and methionine salvage flux were not measured in this study, and no cellular or animal model was used. Figure 5 therefore depicts an association-based hypothesis generated by the metabolomic data, not an experimentally validated causal pathway.

## 4. Discussion

This study combined targeted serum metabolomics with explainable machine learning in a cohort of 79 patients with DILI and 221 healthy controls. A 32-metabolite panel selected under a union strategy supported an XGBoost classifier that distinguished pure cholestatic from pure hepatocellular injury, and TreeExplainer-based SHAP attribution identified MTA as the dominant contributor to the CD prediction. A four-parameter logistic fit of the SHAP dependence curve for MTA crossed the decision baseline at a relative abundance of 93. When the full 32-metabolite XGBoost classifier was applied to the 18 clinically labelled MD patients as an independent test set, the resulting multivariate classifications aligned directly with this model-derived MTA transition point. Sixteen of these patients mapped onto the HD trajectory and two onto the CD trajectory at single-patient resolution. To our knowledge, this is the first DILI study to translate a high-dimensional SHAP decision surface into an analytical single-molecule transition point for sub-phenotype evaluation, and the framework complements rather than replaces the R-value with a metabolomics-derived molecular axis.

The clinical category of mixed-pattern DILI, defined empirically by 2 < R < 5 [4,5], is operationally indispensable but pathophysiologically heterogeneous, and the half-life mismatch between ALT and ALP together with operator-dependent RUCAM scoring further blurs its boundaries [10,13]. Because hepatocellular and cholestatic injuries carry distinct chronicity risks and therapeutic implications [9,11], this ambiguity directly affects whether a patient is considered for glucocorticoid-based management of necroinflammatory injury or for ursodeoxycholic-acid-based cholangiopathy care [4,5]. Our data suggest that, within the resolution of the 32-metabolite panel examined here, MD is composed of two metabolic-pattern groups: a majority with a profile resembling hepatocellular injury and a minority (MD_14 and MD_18) with a profile resembling cholestasis. We propose the framework as two tiers. In the first tier, MTA could serve as a candidate molecular adjudication marker around the 93 transition point, with the SHAP surface saturating on a positive plateau above this value and remaining negative below it. In the second tier, the full 32-metabolite XGBoost ensemble could serve as a higher-resolution adjudicator for cases that fall within the 95% confidence band of the 4PL fit. Both tiers require prospective external validation, including model calibration analyses and decision-impact studies, before any clinical role is asserted. Methodologically, our approach aligns with recent advances in hepatology, where SHAP-interpreted gradient boosting is increasingly used for disease reclassification [29]. Clinically, this framework addresses the widely recognised unmet need for sub-phenotype-level resolution in DILI biomarker research [14,15,16,17,21].

Mechanistically, we propose that MTA functions in this setting as a specific marker of biliary excretory failure rather than a generic correlate of hepatocyte rupture. Previous macroscopic profiling of this parental DILI cohort identified 5′-MTA elevation primarily as a generic indicator of redox disruption and disease progression [48]; our high-resolution machine-learning framework redefines its biological utility. Rather than a mere bystander of non-specific injury, we demonstrate that MTA is specifically coupled to cholestatic pathology. This hypothesis is supported by three distinct observations. First, MTA was uncoupled from hepatocellular necrosis markers, with no significant correlation with ALT (r = −0.19, *p* = 0.10) or AST (r = 0.06, *p* = 0.61), and circulating levels in HD patients were statistically indistinguishable from healthy controls (Figure 3A). If MTA were spilling passively from ruptured hepatocytes, the ALT-rich HD group would be expected to track with it, which is not observed here. Second, MTA correlated with total bilirubin (r = 0.53, *p* < 0.001), a recognised clinical marker of biliary excretory dysfunction [45,46,49]. Finally, the SHAP dependence surface for MTA was sigmoidal rather than linear, implying a tipping point in the underlying biology rather than a graded leakage process. Within the methionine salvage architecture, we hypothesise that intrahepatic accumulation of toxic bile acids, the defining lesion of cholestatic DILI [44,45,46], suppresses the activity or expression of MTAP. Because MTA is the obligate upstream substrate of MTAP, generated stoichiometrically as a side-product of polyamine biosynthesis, reduced MTAP flux would create a metabolic bottleneck, allowing MTA to accumulate and spill into the systemic circulation [22,23]. This process closely mirrors the concurrent rise in bilirubin, as both metabolites are driven into the circulation by the shared underlying canalicular impairment. In this context, we speculate that the transition point of 93 corresponds to the abundance at which residual MTAP recycling capacity is exceeded. This is consistent with established evidence that flux through the adjacent MAT/SAM node of the methionine cycle regulates hepatic injury and mitochondrial homeostasis [50,51]. Importantly, the present study measured circulating metabolites only. MTAP expression, enzymatic activity, polyamine flux, and methionine salvage turnover were not assessed, nor were any perturbation experiments performed. Therefore, our data demonstrate a statistical association between MTA abundance and the cholestatic phenotype, rather than a causal role for the MTA–MTAP axis. Consequently, this axis must be regarded strictly as a hypothesis generated by these findings, rather than an experimentally supported mechanism.

Within the broader DILI biomarker landscape, established indicators such as keratin-18 and glutamate dehydrogenase primarily reflect the magnitude of hepatocyte necrosis or apoptosis, tracking closely with aminotransferases [17,43]. By contrast, MTA demonstrated a distinct clinical pattern in our cohort. It was completely uncoupled from aminotransferases, showing no elevation in HD patients despite a ~50-fold median increase in ALT, and instead correlated preferentially with total bilirubin. Consequently, rather than replacing necrosis markers, MTA serves as a complementary indicator specifically reflecting biliary excretory impairment. Although a direct discriminatory benchmark was precluded because keratin-18 and glutamate dehydrogenase were not measured in this retrospective cohort, evaluating a combined panel of necrosis and cholestasis markers represents a critical objective for future prospective validation.

To ensure methodological robustness, our analysis was explicitly designed to prioritise interpretability. TreeExplainer was utilised because it computes exact, rather than approximate, Shapley values for tree ensembles [26]. By distilling the high-dimensional SHAP surface into a single-metabolite dependence curve, the model’s decision boundary is rendered as an interpretable biochemical transition rather than an abstract feature-importance ranking. This approach aligns with broader advancements across computational biomedicine. Data-driven models with attribution layers are widely used for clinical outcome prediction [28,29]. In a parallel domain, structure-based pipelines employ virtual screening and molecular dynamics to evaluate physical binding hypotheses, such as identifying multitargeted inhibitors for cervical cancer [52]. Both methodologies share a fundamental requirement for rigour: computational predictions must terminate in experimentally testable hypotheses. Whether evaluating a specific protein–ligand interaction or isolating a metabolic bottleneck, our framework satisfies this demand for transparent, biologically actionable outputs.

The principal limitation of this study is the small number of pure cholestatic cases (n = 7). While reflecting the epidemiological scarcity of this phenotype in TCM-associated DILI [8,21], it inevitably constrains our downstream inferences. With only six cholestatic patients per training partition, the classifier learns from a minority class whose biological diversity (e.g., age, culprit herb, comorbidity, severity) is naturally narrower than that of the broader cholestatic population. Although LOOCV with within-fold SMOTE mitigates resampling bias, it cannot manufacture unobserved variability. Because SMOTE strictly interpolates within the convex hull of observed minority samples, it merely densifies the known feature space rather than capturing unseen phenotypes [37,40]. Consequently, performance estimates from small samples remain unstable and potentially optimistic [53]. The LOOCV AUC of 0.950 and the transition point of 93 must therefore be interpreted as cohort-conditioned estimates rather than robust clinical metrics. We explicitly emphasise that this transition point is exploratory and must not be used as a clinical decision limit pending the external validation outlined below.

Furthermore, the cohort was restricted by design to DILI attributable to traditional Chinese medicine (TCM). This deliberate restriction improves internal consistency by eliminating between-drug-class metabolic variance, a recognised confounder given the established metabolic heterogeneity across different DILI aetiologies. While herbal products and conventional pharmaceuticals possess different upstream hepatotoxic mechanisms, the proposed MTA–MTAP bottleneck sits downstream of intrahepatic bile-acid accumulation—a universal pathological feature of cholestatic injury. Therefore, extrapolating the MTA transition point to cholestatic DILI caused by conventional drugs is biologically plausible. Nevertheless, clinical validation in independent cohorts encompassing non-TCM aetiologies remains essential to definitively establish its external generalisability.

Demographic and clinical heterogeneity introduce important confounding factors. The healthy reference group was younger (32.3 ± 10.3 years) and predominantly male (84.6%), whereas the DILI groups were older (48.0–56.0 years) and predominantly female (16.7–35.2% male; Table 1). Given that age and sex strongly influence the circulating metabolome [54,55], the initial healthy-versus-DILI contrasts inevitably carry demographic components. Crucially, this confounding is mitigated in the core of our analysis. The XGBoost classifier, SHAP attribution, and 4PL transition point were derived entirely within the DILI cohort (HD vs. CD), where age and sex distributions are highly comparable. Nevertheless, considering the remaining clinical heterogeneity of DILI (e.g., comedication and nutritional status) and the limited cholestatic sample size, the 32-metabolite panel and MTA transition point should be robustly viewed as candidate findings requiring independent validation.

The specificity of the MTA signal must be evaluated within a broader hepatological context. MTAP perturbation is not unique to acute cholestatic injury. Reduced abundance of methionine metabolism enzymes occurs in human liver cirrhosis and hepatocellular carcinoma [56]. Furthermore, promoter hypermethylation silences MTAP in numerous hepatocellular carcinomas [57], and homozygous MTAP deletion across solid tumours raises circulating MTA [47]. Consequently, elevated MTA represents a shared readout of impaired methionine salvage under various hepatic and neoplastic stresses rather than a uniquely cholestatic defect. However, specific design features ensure our findings are not confounded by this chronic-disease background. Patients with cirrhosis, chronic viral hepatitis, fatty liver disease, hereditary metabolic disorders, and malignancies were strictly excluded at enrolment. Additionally, our discriminating contrast is internal to the DILI cohort (cholestatic versus hepatocellular pattern) rather than a comparison against healthy controls.

From an analytical perspective, converting this relative-abundance transition point into a clinically actionable measurement requires a rigorous analytical transition. The value of 93 represents a ratio of analyte to internal-standard response under the specific extraction, chromatography, ionisation, and normalisation conditions of our assay. As inter-laboratory studies demonstrate, even well-standardised targeted metabolomics platforms require explicit harmonisation before cross-site comparison [58]. Translating this metric to an absolute serum concentration entails a five-step bioanalytical sequence: (i) procurement of a stable-isotope-labelled MTA internal standard, spiked at a fixed concentration into all samples; (ii) construction of a matrix-matched calibration curve using authentic unlabelled MTA in stripped or surrogate serum across physiological and pathological ranges; (iii) comprehensive quantitative method validation adhering to accepted bioanalytical guidelines [32] (covering linearity, lower limit of quantification, intra- and inter-day accuracy and precision, recovery, matrix effect, and stability); (iv) establishment of age- and sex-stratified reference intervals in a healthy population, acknowledging the demographic dependence of the circulating metabolome [54,55]; and (v) re-derivation of the decision threshold in absolute concentration units within an independent, adequately powered cohort, utilising a pre-specified statistical analysis plan. Only after step (v) can a concentration-based cutoff be established. The value of 93 reported here cannot be arithmetically converted into a concentration, nor should it be applied as a threshold in other laboratories.

These limitations converge on a single requirement: prospective, fully independent external validation. The necessary study should be multicentre, enrolling consecutive patients with DILI of mixed aetiology. A target of at least 50 confirmed cholestatic and 50 confirmed hepatocellular cases is required to re-estimate the transition point with a reliable confidence interval. Phenotype should be assigned by R-value at the first qualifying laboratory set, accompanied by blinded RUCAM adjudication and, where clinically indicated, histological confirmation within the mixed-pattern stratum. Until such validation is complete, MTA remains a candidate metabolic indicator supported by hypothesis-generating evidence, and the MTA-MTAP axis constitutes a mechanistic hypothesis awaiting experimental confirmation.

## 5. Conclusions

In conclusion, by integrating targeted metabolomics with explainable machine learning, this study translates high-dimensional biochemical data into an interpretable molecular framework for DILI sub-phenotyping. We identify MTA as a candidate metabolic indicator that may help resolve the clinical ambiguity of mixed-pattern DILI. The correlation of MTA with total bilirubin, coupled with its dissociation from ALT, supports a specific excretory impairment mechanism rather than generic hepatocellular necrosis. Building on this, the proposed MTA-MTAP axis provides a mechanistic hypothesis for future experimental investigations into cholestatic injury. While the model-derived transition point reported here remains an exploratory estimate requiring prospective validation in independent cohorts, this approach demonstrates that empirical clinical indices can be effectively complemented by mechanistically relevant molecular markers, providing a biological basis for precision phenotyping in DILI.

## Figures and Tables

**Table 1:** Baseline demographic and clinical characteristics of the study cohorts.

Characteristics	Healthy (n = 221)	HD Phenotype (n = 54)	CD Phenotype (n = 7)	MD Phenotype (n = 18)
Demographics				
Age (years), mean ± SD	32.3 ± 10.3	48.0 ± 13.7	56.0 ± 17.9	51.3 ± 13.6
Male gender, *n* (%)	187 (84.6%)	19 (35.2%)	2 (28.6%)	3 (16.7%)
Liver Biochemistries				
ALT (U/L), median (IQR)	16.0 (12.0–21.0)	894.2 (666.6–1248.4)	39.3 (34.0–216.4)	172.8 (127.0–218.8)
AST (U/L), median (IQR)	20.0 (18.0–22.0)	617.0 (379.0–895.0)	69.0 (49.4–183.2)	253.9 (154.5–520.9)
ALP (U/L), median (IQR)	68.0 (58.0–80.0)	120.0 (103.5–173.0)	189.1 (134.2–209.0)	122.4 (106.8–209.5)
TBIL (μmol/L), median (IQR)	13.5 (10.5–18.0)	111.7 (48.5–217.7)	239.6 (124.0–318.3)	46.3 (32.6–168.1)
R-value, median (IQR)	N/A	19.1 (10.2–30.5)	1.2 (0.6–1.6)	3.7 (2.9–4.2)

N/A, not applicable. The R-value is a specific diagnostic index used to classify the pattern of liver injury and is clinically meaningless for healthy individuals with normal baseline biochemistries.

## Data Availability

Data are contained within the article or Supplementary Materials. The original contributions presented in this study are included in the article/Supplementary Materials. Further inquiries can be directed to the corresponding author.

## Supplementary Materials

The following supporting information can be downloaded at: https://www.mdpi.com/article/10.3390/metabo16090614/s1. Supplementary Methods S1: Detailed LC-MS/MS protocol; Supplementary Methods S2: Derivation of the 4PL transition point and Monte Carlo confidence intervals for MTA; Supplementary Methods S3: XGBoost Hyperparameter Policy; Table S1: Differential serum metabolites selected under the union strategy; Table S2: Algorithm configuration of the XGBoost classifier; Table S3: Spearman correlation coefficients between the differential metabolites and five clinical liver-injury indices; Table S4: Spearman correlation *p* values for the differential metabolites against five clinical liver-injury indices; Table S5: Per-patient covariates, MTA abundance, predicted *P*(CD) and metabolic trajectory assignment for the 18 MD patients.

## Abbreviations

CD, cholestatic; DILI, drug-induced liver injury; HD, hepatocellular; LC-MS/MS, liquid chromatography-tandem mass spectrometry; LOOCV, Leave-One-Out Cross-Validation; MD, mixed-pattern; MTA, 5′-methylthioadenosine; MTAP, methylthioadenosine phosphorylase; OPLS-DA, orthogonal partial least squares discriminant analysis; RUCAM, Roussel Uclaf Causality Assessment Method; SAM, S-adenosyl-L-methionine; SHAP, SHapley Additive exPlanations; SMOTE, Synthetic Minority Over-sampling Technique; TBIL, total bilirubin; TCM, traditional Chinese medicine; ULN, upper limit of normal; 4PL, four-parameter logistic; AUC, area under the receiver operating characteristic curve; VIP, variable importance in projection; XGBoost, eXtreme Gradient Boosting.
