# Supplementary material for: Metabolomics and Machine Learning Uncover 5′-Methylthioadenosine as a Metabolic Indicator of Mixed-Pattern Drug-Induced Liver Injury
Source: Metabolites. 2026 Aug 27;16(9):614. doi: 10.3390/metabo16090614 (PMC13609085; doi:10.3390/metabo16090614)
Supplement: Supplementary file 1 [file metabolites-16-00614-s001.zip › metabolites-4499944-supplementary.pdf]

Supplementary Materials

Metabolomics and Machine Learning Uncover 5′-Methylthioadenosine as a Metabolic Indicator of Mixed-Pattern Drug-Induced Liver Injury

Supplementary Methods S1. Detailed LC-MS/MS protocol

MS-grade acetonitrile and HPLC-grade isopropyl alcohol were obtained from Thermo Fisher Scientific (Pittsburgh, PA, USA); formic acid was obtained from TEDIA (Fairfield, OH, USA); ultrapure water was produced on a Milli-Q Direct 8 system (Millipore, Bedford, MA, USA). The internal-standard (IS) mixture (Cambridge Isotope Laboratories, Cambridge, MA, USA) comprised eight stable-isotope-labelled compounds, thymine-d4, valine-d8, phenylalanine-d8, 17-hydroxyprogesterone-d8, docosahexaenoic acid-d5, cholic acid-d4, chenodeoxycholic acid-d4 and glycocholic acid-d4, each at 400 ng/mL.

Sample preparation. Fifty microlitres of serum were combined with 140 µL of cold methanol and 10 µL of the IS mixture, vortexed for 2 min (Vortex-Genie 2, Scientific Industries, Bohemia, NY, USA) and centrifuged at 13,800 rpm for 10 min at 4 °C (Heraeus Fresco 21, Thermo Scientific, Waltham, MA, USA). The supernatant was transferred to autosampler vials for LC-MS/MS injection.

Liquid chromatography. Chromatographic separation was performed on a Spark Holland LC system (Emmen, The Netherlands) using an XSelect HSS T3 column (150 × 2.1 mm, 3.5 µm; Waters, Milford, MA, USA) at a column-oven temperature of 20 °C with a flow rate of 0.5 mL/min and an injection volume of 5 µL. Mobile phase A was water containing 0.1 % formic acid; mobile phase B was acetonitrile/isopropyl alcohol (7:2, v/v). The gradient was 1–10 % B (0–4 min), 10–50 % B (4–8 min), 50–80 % B (8–15 min), 80–100 % B (15–25 min), 100 % B (25–27 min), 1 % B (27–27.1 min) and 1 % B re-equilibration (27.1–29 min).

Mass spectrometry. Detection used an AB Sciex API 5500 triple-quadrupole mass spectrometer with a Turbo Ion Spray source (Concord, Canada) operated in scheduled multiple-reaction-monitoring (sMRM) mode in both polarities within a single injection. The source temperature was 600 °C; gas 1, gas 2 and curtain gas were 60, 60 and 40 psi, respectively; the ESI capillary voltages were –4500 V in negative mode and +5500 V in positive mode. The targeted panel comprised 143 metabolites.

Quality control and data processing. Pooled-serum quality-control samples were interspersed at 1-in-20 injections. Raw data were processed in Multi-Quant v3.0.2 with weighted least-squares ($1/x^2$) calibration. Inter-day relative standard deviations in pooled-serum QCs were below 20 % for all metabolites.

Supplementary Methods S2. Derivation of the 4PL Transition Point and Monte Carlo confidence intervals for MTA

We modelled the non-linear relationship between the relative abundance of 5′-methylthioadenosine (MTA) and its SHAP value (the marginal contribution to the log-odds of the cholestatic prediction) by a four-parameter logistic (4PL) regression. Parameter estimation and uncertainty propagation were implemented in Python using the scipy.optimize and numpy libraries.

1. Formulation. Let x denote the relative abundance of MTA and y the corresponding SHAP value. The dependence was mathematically defined as:

$$y=\frac{L}{1+\exp\left(-k(x-x_0)\right)}+b$$

where L denotes the upper-plateau span, k represents the slope at the inflection point, x_0 is the inflection abundance, and b is the lower-plateau offset.

$$\hat{\theta} = (\hat{L}, \hat{k}, \hat{x}_0, \hat{b})$$

2. Levenberg–Marquardt optimisation. The optimal parameter vector $\hat{\theta} = (\hat{L}, \hat{k}, \hat{x}_0, \hat{b})$ and its estimated covariance matrix Σ were obtained via non-linear least-squares optimisation using the Levenberg–Marquardt algorithm (scipy.optimize.curve_fit). Initial parameter estimates were data-driven: $L_{init} \approx \max(y) - \min(y)$, $x_{0,init} \approx \mathrm{median}(x)$, $k_{init} \approx 0.1$ and $b_{init} \approx \min(y)$. A strict tolerance was used with a maximum of 10,000 function evaluations and convergence was checked against the residual sum of squares and the parameter increment norm.

3. Analytical transition point. The model-derived transition point corresponds to the abundance at which the metabolic signature transitions from a negative to a positive contribution to the CD prediction, that is the value of x at which y=0. This value was obtained analytically by inverting the 4PL equation:

$$x_{threshold} = \hat{x}_0 - \frac{1}{\hat{k}} \ln\left(\frac{\hat{L}}{-\hat{b}} - 1\right)$$

Substituting $\hat{\theta}$ yielded $x_{threshold} = 93$ in relative abundance units.

4. Monte Carlo confidence band. To construct a 95 % confidence band around the fitted curve we drew $N = 1000$ independent parameter vectors $\theta_i \sim N(\hat{\theta}, \Sigma)$. For a uniformly spaced grid of abundance values x_j spanning the empirical data range we computed an ensemble of simulated SHAP trajectories $y_{i,j} = f(x_j; \theta_i)$. The 95 % envelope at each x_j was the 2.5th and 97.5th percentile of $y_{i,j}$. This procedure propagates the parameter covariance of the optimised fit into the prediction space.

Supplementary Methods S3. XGBoost Hyperparameter Policy

All XGBoost hyperparameters listed in Supplementary Table S2 were fixed before any cross-validation was executed and were held constant and identical across all 61 LOOCV folds. No fold-specific tuning, nested cross-validation, grid search or randomised search was performed at any stage. The only quantities estimated from data inside each fold were therefore the z-score scaling parameters, the SMOTE synthetic points and the boosted-tree ensemble itself, each derived from the 60 training samples alone. This policy was adopted deliberately: in a cohort of this size, fold-wise tuning would add a

model-selection step whose variance cannot be estimated from the same data and is a recognised source of optimistically biased cross-validated performance.

Supplementary Table S1. Differential serum metabolites selected under the union strategy

Metabolite	H vs DILI		H vs HD		H vs MD		H vs CD	
	VIP	log_2 FC	VIP	log_2 FC	VIP	log_2 FC	VIP	log_2 FC
Taurine	1.13	-1.55	1.19	-1.52	1.14	-1.52	1.17	-1.94
Choline	1.08	-1.00	1.15	-1.15	0.64	-0.80	0.90	-0.60
Niacinamide	1.01	1.18	0.97	1.19	1.08	1.20	1.18	1.05
Glutamine	1.13	-1.08	1.23	-1.15	0.99	-0.94	1.09	-0.97
Histidine	1.05	-0.68	1.12	-0.74	0.93	-0.53	0.91	-0.64
Proline	1.01	-0.51	0.98	-0.59	0.84	-0.26	0.55	-0.63
Phenylalanine	1.15	-0.47	1.13	-0.53	1.02	-0.28	0.82	-0.52
Isoleucine	1.08	-0.58	1.05	-0.60	1.12	-0.40	0.80	-0.90
Tyrosine	1.11	0.34	0.71	0.23	0.99	0.63	1.15	0.40
Succinate	1.05	0.63	0.88	0.71	0.88	0.54	1.09	0.19
5′-Methylthioadenosine	1.83	2.79	0.61	1.34	1.41	2.83	0.81	5.34
S-Adenosyl-L-methionine	1.54	4.58	1.64	4.43	2.23	4.70	2.06	5.23
Phosphoethanolamine	1.12	2.40	1.15	2.47	1.07	2.43	1.50	1.64
AMP	1.14	7.67	1.22	7.73	1.60	7.67	1.63	7.14
Aspartate	1.02	-0.84	1.08	-1.00	0.53	-0.75	0.87	-0.12
Pyruvate	1.16	2.02	1.13	2.14	1.51	1.85	1.77	1.35
Acetyl-L-carnitine	1.02	0.02	0.71	-0.32	1.12	0.47	0.80	0.78

Valine	1.09	-0.52	1.12	-0.55	1.15	-0.39	0.88	-0.68
Leucine	1.08	-0.60	1.06	-0.62	1.13	-0.45	0.83	-0.91
Xanthine	1.10	-1.87	1.22	-1.98	0.92	-1.82	1.19	-1.28
GMP	1.14	5.23	1.20	5.34	1.76	5.07	1.50	4.75
Betaine hydrochloride	1.03	0.82	0.82	0.81	0.50	1.02	1.03	0.26
Hypoxanthine	1.21	-3.52	1.35	-3.89	1.16	-3.08	1.33	-2.65
Cysteine	1.05	2.27	1.02	2.18	1.45	2.61	1.62	1.92
Malic acid	1.29	2.81	1.42	2.79	1.77	2.99	1.73	2.31
Uracil	1.13	-1.22	1.25	-1.29	1.08	-1.02	1.12	-1.27
Pantothenate	1.16	-0.30	0.62	-0.36	1.16	-0.35	0.58	0.91
Hydroxyproline	1.09	-0.56	1.06	-0.59	1.13	-0.39	0.80	-0.91
Creatinine	1.37	1.04	1.49	1.01	1.86	1.17	1.78	0.95
Uridine	1.12	-1.23	1.22	-1.28	1.05	-1.07	1.11	-1.27
N-Acetylornithine	1.07	-1.12	1.11	-1.37	0.42	-1.22	1.04	0.14
Xanthosine	1.09	-2.22	1.19	-2.46	0.82	-1.94	1.04	-1.51

Supplementary Table S2. Algorithm configuration of the XGBoost classifier.

Parameter	Value
Algorithm	eXtreme Gradient Boosting (XGBoost)
objective	binary:logistic
eval_metric	logloss
learning_rate	0.05
max_depth	3
n_estimators	50
min_child_weight	1
gamma	0

subsample	1.0
colsample_bytree	0.80
reg_alpha	0
reg_lambda	1
random_state	88
Cross-validation	Leave-One-Out Cross-Validation (LOOCV)
Resampling	SMOTE (imblearn.over_sampling.SMOTE, k_neighbors = 2, applied exclusively within the training partition of each LOOCV iteration)
Training cohort	Real polar cohort (HD = 54, CD = 7; n = 61)
Held-out independent test	MD cohort (n = 18; not used in training, SMOTE, hyper-tuning or SHAP fitting)

Supplementary Table S3. Spearman correlation coefficients between the differential metabolites and five clinical liver-injury indices

Metabolite	ALT (necrosis)	AST (necrosis)	ALP (cholestasis)	TBIL (cholestasis)	R-value
Taurine	0.09	0.01	-0.07	0.02	0.08
Choline	-0.18	0.06	0.14	0.28	-0.20
Niacinamide	-0.02	0.03	0.03	0.10	-0.07
Glutamine	-0.04	0.08	-0.10	0.25	-0.01
Histidine	-0.14	0.00	-0.03	0.20	-0.12
Proline	-0.10	0.14	0.03	0.37	-0.11
Phenylalanine	-0.16	0.13	0.06	0.43	-0.16
Isoleucine	0.15	0.25	-0.16	-0.03	0.15
Tyrosine	-0.14	0.18	-0.01	0.47	-0.12
Succinate	0.15	0.11	-0.02	0.13	0.11
5′-Methylthioadenosine	-0.19	0.06	0.21	0.53	-0.21

S-Adenosyl-L-methionine	-0.23	-0.04	0.04	0.32	-0.23
Phosphoethanolamine	0.16	0.04	-0.05	-0.06	0.13
AMP	0.17	0.07	0.04	0.06	0.12
Aspartate	-0.09	-0.04	0.01	0.23	-0.11
Pyruvate	0.28	0.35	-0.09	0.25	0.28
Acetyl-L-carnitine	-0.19	-0.01	-0.19	0.23	-0.10
Valine	0.03	0.21	-0.12	0.00	0.03
Leucine	0.18	0.27	-0.17	-0.05	0.18
Xanthine	-0.27	-0.11	0.02	0.44	-0.22
GMP	0.16	-0.01	0.05	-0.14	0.11
Betaine hydrochloride	0.12	0.34	0.02	0.47	0.10
Hypoxanthine	-0.26	-0.18	0.05	0.22	-0.25
Cysteine	-0.06	-0.06	-0.22	-0.47	0.01
Malic acid	0.15	0.24	-0.08	0.24	0.15
Uracil	-0.13	0.03	0.00	0.12	-0.14
Pantothenate	-0.05	-0.07	-0.01	0.33	-0.06
Hydroxyproline	0.14	0.26	-0.17	0.00	0.15
Creatinine	-0.13	0.08	-0.03	0.26	-0.12
Uridine	-0.07	0.07	0.03	0.16	-0.09
N-Acetylornithine	-0.34	-0.13	0.01	0.04	-0.31
Xanthosine	-0.14	0.12	0.15	0.58	-0.18

Supplementary Table S4. Spearman correlation *P* values for the differential metabolites against five clinical liver-injury indices

Metabolite	ALT (necrosis)	AST (necrosis)	ALP	TBIL	R-value

			(cholestasis)	(cholestasis)	
Taurine	0.428	0.912	0.543	0.829	0.507
Choline	0.110	0.602	0.204	0.013	0.072
Niacinamide	0.867	0.827	0.780	0.379	0.538
Glutamine	0.702	0.506	0.363	0.028	0.920
Histidine	0.219	0.988	0.811	0.074	0.293
Proline	0.361	0.207	0.794	0.001	0.335
Phenylalanine	0.167	0.251	0.599	< 0.001	0.153
Isoleucine	0.177	0.028	0.147	0.824	0.178
Tyrosine	0.222	0.115	0.956	< 0.001	0.312
Succinate	0.196	0.352	0.854	0.244	0.335
5′-Methylthioadenosine	0.098	0.611	0.069	< 0.001	0.059
S-Adenosyl-L-methionine	0.044	0.718	0.699	0.005	0.037
Phosphoethanolamine	0.164	0.715	0.633	0.580	0.261
AMP	0.142	0.534	0.743	0.589	0.308
Aspartate	0.455	0.730	0.939	0.041	0.324
Pyruvate	0.012	0.002	0.438	0.028	0.013
Acetyl-L-carnitine	0.096	0.933	0.094	0.043	0.373
Valine	0.784	0.066	0.305	0.974	0.788
Leucine	0.121	0.016	0.141	0.694	0.122
Xanthine	0.014	0.346	0.889	< 0.001	0.046
GMP	0.167	0.912	0.667	0.206	0.355
Betaine hydrochloride	0.273	0.002	0.836	< 0.001	0.363
Hypoxanthine	0.023	0.121	0.662	0.055	0.024
Cysteine	0.596	0.588	0.047	< 0.001	0.942
Malic acid	0.176	0.032	0.508	0.031	0.187

Uracil	0.247	0.798	0.992	0.286	0.216
Pantothenate	0.637	0.558	0.962	0.003	0.611
Hydroxyproline	0.202	0.023	0.137	0.985	0.191
Creatinine	0.248	0.514	0.809	0.021	0.309
Uridine	0.521	0.525	0.823	0.165	0.429
N-Acetylornithine	0.002	0.247	0.959	0.726	0.006
Xanthosine	0.231	0.313	0.178	< 0.001	0.118

Supplementary Table S5. Per-patient covariates, MTA abundance, predicted *P*(CD) and metabolic trajectory assignment for the 18 MD patients

Patient ID	Age	Sex	ALT (U/L)	AST (U/L)	ALP (U/L)	TBIL (μmol/L)	Baseline R-value	MTA Relative Abundance	Predicted *P*(CD)	Metabolic Trajectory Assignment
MD_01	47	female	158	222	123	37	3.21	3.82	0.041	HD Trajectory
MD_02	79	female	123	226	112	233	3.71	1.47	0.022	HD Trajectory
MD_03	47	male	124	359	122	33	2.55	15.17	0.03	HD Trajectory
MD_04	53	male	110	228	101	55	2.72	6.58	0.03	HD Trajectory
MD_05	59	female	188	544	226	37	2.8	29.31	0.028	HD Trajectory
MD_06	43	male	261	280	160	417	4.09	84.2	0.061	HD Trajectory
MD_07	61	female	285	325	258	32	3.73	3.97	0.018	HD Trajectory
MD_08	36	female	221	215	139	28	3.98	66.89	0.041	HD Trajectory

MD_09	63	female	221	58	275	34	2.71	2.49	0.038	HD Trajectory
MD_10	61	female	213	79	269	27	2.68	2.78	0.027	HD Trajectory
MD_11	53	female	97	50	78	17	4.2	1.58	0.021	HD Trajectory
MD_12	26	female	131	35	93	22	3.52	2.28	0.046	HD Trajectory
MD_13	38	female	196	588	109	46	4.48	9.6	0.031	HD Trajectory
MD_14	34	female	134	614	109	302	3.08	143.74	0.908	CD Trajectory
MD_15	62	female	141	134	106	389	4.49	14.6	0.047	HD Trajectory
MD_16	66	female	126	452	102	138	4.17	7.63	0.023	HD Trajectory
MD_17	57	female	587	755	406	55	4.88	20.16	0.019	HD Trajectory
MD_18	39	female	191	1252	124	168	3.85	221.81	0.961	CD Trajectory
